# Prognostic value of BAP1 expression in uveal melanoma: a comparative study with histopathological factors in a large Spanish cohort

**DOI:** 10.3389/fonc.2026.1842781

**Published:** 2026-05-13

**Authors:** Patricia Valencia Nieto, Ciro García Álvarez, María Elena García Lagarto, María Fe Muñoz Moreno, Patricia Diezhandino García, María Antonia Saornil Álvarez

**Affiliations:** 1 Intraocular Tumours Unit, Valladolid University Clinical Hospital, Valladolid, Spain; 2 Radiation Oncology Department, Valladolid University Clinical Hospital, Valladolid, Spain; 3 Ophthalmology Department, Valladolid University Clinical Hospital, Valladolid, Spain; 4 Histopathology Department, Valladolid University Clinical Hospital, Valladolid, Spain; 5 Research Unit, Valladolid University Clinical Hospital, Valladolid, Spain

**Keywords:** BAP1, inmunohistochemestry, metastasis, prognosis, survival analysis, uveal melanoma

## Abstract

**Purpose:**

To evaluate the prognostic significance of BRCA1-associated protein 1 (BAP1) nuclear expression in enucleated eyes with uveal melanoma (UM), and to compare its predictive value with classical clinicopathological factors in a large Spanish cohort.

**Design:**

Prospective, single-center, observational-cohort study.

**Subjects:**

A total of 150 consecutive patients diagnosed with posterior uveal melanoma and treated with primary enucleation at a national reference center between 2006 and 2024.

**Methods:**

Immunohistochemical analysis of BAP1 expression was performed on formalin-fixed, paraffin-embedded tumor sections. Nuclear staining was classified as low grade (<33% positive tumor nuclei) or high grade (≥33%). Clinicopathological features and survival outcomes were assessed, and multivariate Cox regression was used to identify independent prognostic factors for melanoma-specific survival.

**Main Outcome Measures:**

Melanoma-specific survival and its association with BAP1 nuclear expression and classical prognostic variables.

**Results:**

Low grade nuclear BAP1 expression was detected in 50% of tumors and was significantly associated with worse melanoma-specific survival (hazard ratio [HR] = 2.72; 95% confidence interval [CI]: 1.04–7.11; p = 0.041). Additional independent predictors of poor prognosis included vortex vein invasion (HR = 4.55; p = 0.028), extrascleral extension (HR = 4.73; p = 0.004), and epithelioid cell type (HR = 4.12; p = 0.019). No significant association was observed between BAP1 expression and sex, mitotic index, or Ki-67 proliferation rate.

**Conclusions:**

Low grade nuclear BAP1 immunostaining is a strong, independent predictor of poor prognosis in uveal melanoma, comparable to established histopathological features. Immunohistochemical assessment of BAP1 is a cost-effective tool for risk stratification in routine clinical practice.

## Introduction

Uveal melanoma (UM) is the most common primary intraocular cancer in adults and is characterised by a high metastatic potential despite effective local treatment (1). Worldwide, the incidence ranges from 4.3 to 10.9 cases per million per year (2, 3), with variations between geographical locations.

Although only 1-4% of patients have detectable metastases when diagnosing the primary tumour, up to 50% will develop metastatic disease within the following 15 years, even with successful local treatment (4, 5), highlighting the critical need for accurate prognostic stratification.

Clinical and histopathological risk factors must be considered to better predict metastatic risk (6–9).

Prognostic factors that have been shown to predict the occurrence of metastases and therefore influence prognosis include clinical characteristics such as tumour size, ciliary body invasion, and extrascleral extension (7, 10, 11). In addition to clinical factors, patient characteristics such as Caucasian race, fair skin and light eye colour have also been identified as significant prognostic factors (12). On the other hand, histopathological characteristics such as size, cell type, mitotic index, and lymphocytic infiltration have also been related to the development of metastases (13, 14). In a large Spanish series of enucleated eyes (9), the histopathological characteristics associated with poorer survival were high Ki-67 expression (≥ 15%; p = 0.037), vortex vein invasion (p < 0.001), emissary canal invasion (p = 0.005), extrascleral extension (p = 0.001) and low percentage of necrosis (< 10%).

More recently, genetic and molecular alterations have been recognized as powerful, independent prognostic factors. Harbour et al. (15) showed that class 2 GEP (Gene Expression Profile) correlates with mutations in the BRCA1-associated protein 1 tumour suppressor gene, BAP1. The BAP1 gene was first described in 1998 by Jensen (16) and is located on chromosome 3p21.1, which is frequently deleted in uveal melanoma.

The BAP1 gene encodes a nuclear deubiquitinase that catalyses the removal of individual nuclear ubiquitin chains involved in the epigenetic modulation of chromatin (17). The BAP1 protein is normally localised to the nucleus due to the presence of the nuclear localisation sequence (NLS). In the presence of a ubiquitin-conjugating enzyme (UBE20), the NLS is coated with ubiquitin, which localises BAP1 protein to the cytoplasm of the cell. The wild-type BAP1 protein can remove ubiquitin and thus re-enter the nucleus. The mutant BAP1 protein loses its self-ubiquitylation ability, so it remains in the cytoplasm and loses its tumour suppressor activity (18, 19).

Despite its strong biological rationale, the incremental prognostic value of BAP1 immunohistochemistry compared with established clinical and histopathological prognostic factors remains insufficiently defined in large, real-world cohorts.

Beyond somatic mutations in UM, germline BAP1 mutations define the BAP1 tumour predisposition syndrome (BAP1-TPDS, OMIM #614327), associated with mesothelioma, cutaneous melanoma, meningioma, lung adenocarcinoma, and renal cell carcinoma (20, 21). This autosomal dominant syndrome has high penetrance (>80%) and is characterised by early-onset, aggressive tumours with increased metastatic potential and reduced survival (22–24).

The assessment of BAP1 status via immunohistochemistry (IHC) has shown high concordance with mutation status, offering a cost-effective alternative to molecular testing (25). Despite these findings, data on BAP1 expression in Spanish UM cohorts remain scarce.

In a previously published study from our group (9), classical histopathological prognostic factors were comprehensively analysed, identifying high Ki-67 expression, vortex vein and emissary canal invasion, extrascleral extension, and low necrosis percentage as independent predictors of poor survival. Building upon these findings, the present study aims to evaluate the prognostic significance of nuclear BAP1 expression in a large Spanish series of enucleated uveal melanoma cases, and to compare its predictive value with the classical clinical and histopathological factors previously characterized (9).

## Material and methods

### Patients and clinical assessments

This is a single-centre prospective case series including all consecutive patients diagnosed with posterior uveal melanoma (involving the ciliary body and/or choroid) and treated by enucleation as the primary treatment in the Adult Intraocular Tumour Unit at the University Clinical Hospital of Valladolid between January 2006 and December 2024.

Patients were excluded if they had been followed up for less than 6 months, had been enucleated at another centre, or had insufficient material for histopathological analysis. The diagnosis of uveal melanoma was made by an experienced ocular oncologist (MAS and CGA) based on ophthalmoscopic and ultrasound characteristics. Cases of suspected extraocular extension were assessed by ocular magnetic resonance and ultrasound (26), while the assessment of systemic extension before surgery included a blood liver function test and ultrasound or abdominal computed tomography.

Enucleation was indicated for large tumours (largest diameter > 16 mm of any thickness, > 16 mm basal diameter, > 10 mm apical height, and/or disease categorized as T3/T4 by the TNM classification) (27), as well as tumours with optic nerve invasion, infiltrative growth patterns or not suitable for a conservative approach. Follow-up was scheduled at 1, 3, 6 and 12 months, every 6 months from 1 to 5 years after therapy, and thereafter annually, including systemic assessment (abdominal ultrasound and blood liver function tests).

Date and cause of death (as melanoma or other cause) were recorded.

### Clinical data

Epidemiological and clinical characteristics were prospectively collected in a previously designed, protocol-driven patient registry database. The clinical characteristics assessed were iris colour, tumour laterality, size and shape, location of the anterior margin, scleral extension, and presence of metastasis at diagnosis.

The tumour size was assessed based on the largest tumour diameter (28) and the TNM stage according to the Choroidal and Ciliary Body Melanoma TNM staging system (8th ed., 2017) (29). Although there were three editions of the TNM classification between 2006 and 2021, the TNM 8th edition (29) was used for retrospectively collected data.

### Immunohistochemical BAP1 staining

**Figure 1:**
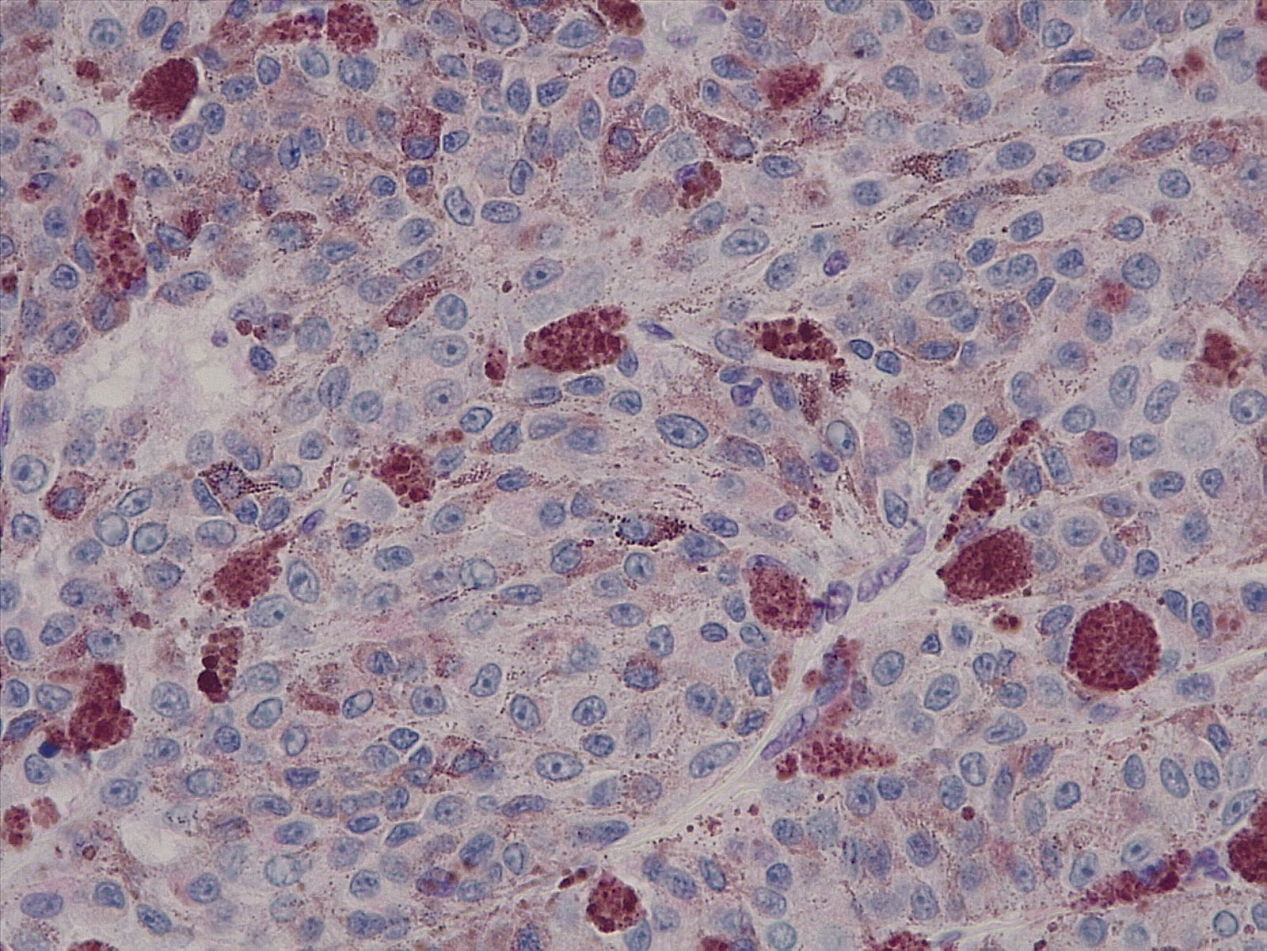
Representative immunohistochemical image of low BAP1 expression in primary uveal melanoma. Fewer than 33% of tumour cell nuclei show positive nuclear staining, with most nuclei lacking BAP1 expression and appearing blue due to hematoxylin counterstaining. Original magnification ×40.

**Figure 2:**
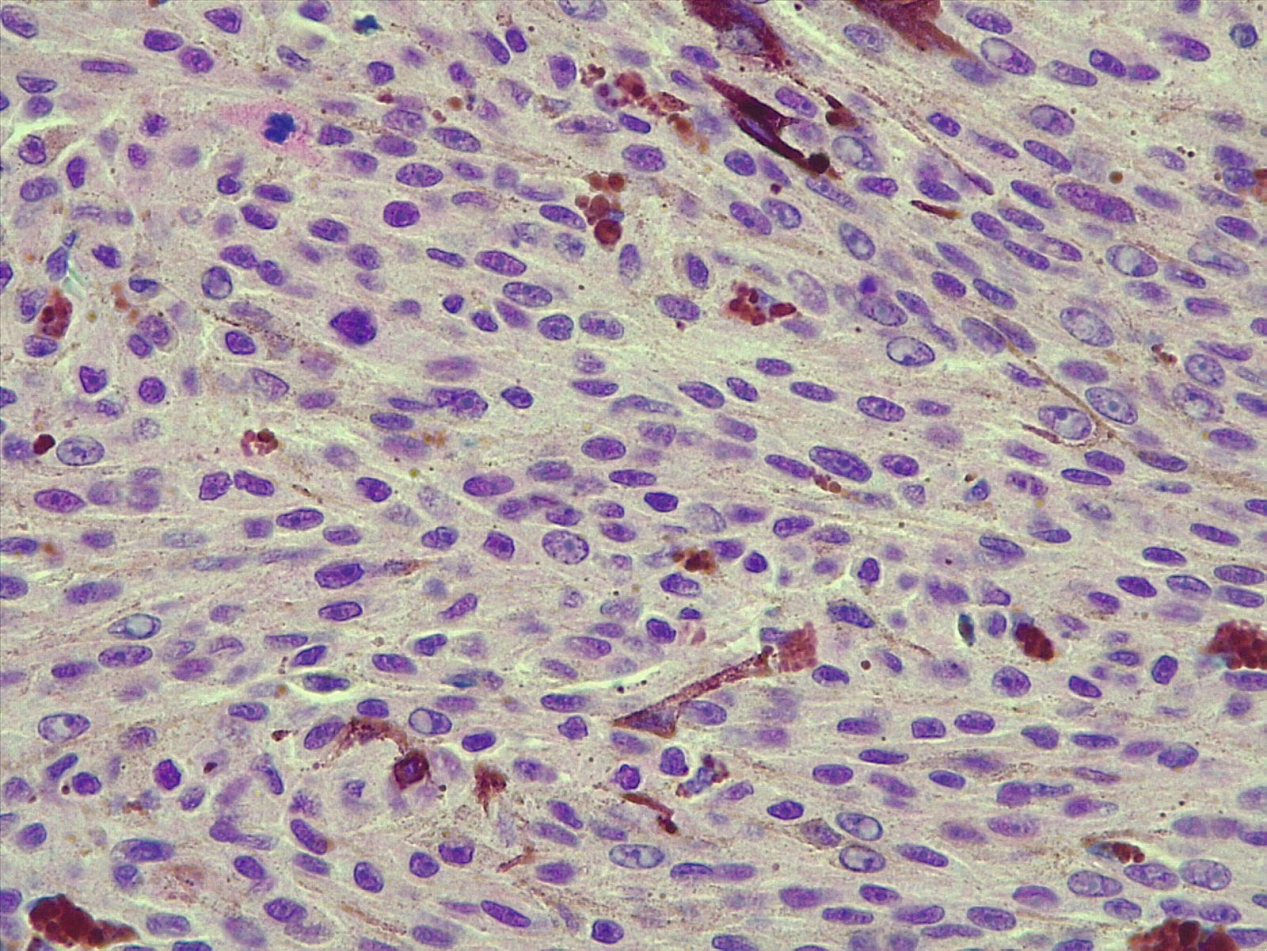
Representative immunohistochemical image of high BAP1 expression in primary uveal melanoma. At least 33% of tumour cell nuclei show positive nuclear staining for BAP1. Original magnification ×40.

Immunohistochemical analysis of BAP1 protein expression was performed on formalin-fixed, paraffin-embedded (FFPE) sections from enucleated uveal melanoma specimens. After standard deparaffinization, antigen retrieval was carried out using EnVision FLEX Target Retrieval Solution High pH (Agilent Technologies) at 97 °C for 30 minutes. Sections were incubated with a primary monoclonal antibody against BAP1 (clone BSB-109) at a dilution of 1:50 for 30 minutes at room temperature. Detection was performed using the EnVision FLEX visualization system (Agilent Technologies), including the EnVision FLEX+ Mouse Linker, followed by diaminobenzidine (DAB) as chromogen. Nuclear immunoreactivity for BAP1 was assessed at high magnification (20× and/or 40× objectives) by two independent observers—a pathologist and an oncologist—both blinded to clinical and pathological data. Positive BAP1 immunoreactivity was indicated by an increase in brown staining in the cell nucleus, whereas negative BAP1 immunoreactivity was characterised by an absence or marked reduction of nuclear staining, resulting in a blue appearance of the nuclei due to hematoxylin counterstain. The level of BAP1 expression was classified as low grade if fewer than 33% of tumour cell nuclei were positive (**Figure 1**), and high grade if 33% or more were positive (**Figure 2**), following the criteria described by Herrspiegel et al. (30). Discrepancies between evaluators were resolved by consensus.

External positive controls included pancreatic tissue samples known to express BAP1. Internal controls within the enucleated specimens comprised normal structures such as the ganglion cell layer of the retina, choroidal melanocytes, and optic nerve nuclei, which exhibited consistent nuclear staining.

### Histopathological analysis

Sections were taken from formalin- fixed paraffin- embedded specimens, deparaffinized, and mounted on glass slides. These sections were then stained with hematoxylin-eosin and evaluated for histopathological characteristics by an experienced ocular pathologist using a protocol based on a previously published methodology (9).

The characteristics analysed were: cell type according to the modified Callender classification system (grouped into spindle A, spindle B, mixed or epithelioid cells for our analysis), Ki-67 expression (recorded as percentage of positively stained cells < or ≥ 15%), vortex vein and/or emissary canal invasion, scleral extension, extent of tumour necrosis (classified as < or ≥ 10% of the tumour area) and BAP1 immunohistochemistry staining, recorded as high or low grade.

### Data collection and statistical analysis

Quantitative variables are expressed as mean ± standard deviation, and qualitative variables as frequencies and percentages. Normality was assessed using the Kolmogorov–Smirnov test.

Associations between categorical variables (sex, age group, eye, iris colour, Ki-67 expression, emissary canal invasion, location of anterior margin, cell type, mitotic count, optic nerve invasion, scleral extension, pigmentation, and necrosis) were analysed using the Pearson chi-square test. When more than 20% of expected cell counts were <5, Fisher’s exact test was applied (e.g., metastasis), while the likelihood ratio test was used for variables with more than two categories (large diameter, AJCC 8th edition stage, COMS classification, tumour shape, and inflammation).

For continuous variables, comparisons between groups were performed using the Student’s t-test for independent samples.

Survival analysis was performed using Kaplan–Meier curves for melanoma-specific survival and overall survival, and differences between groups were assessed using the log-rank test.

Multivariate analysis of prognostic factors for mortality was performed using Cox proportional hazards regression, including variables that were statistically significant in univariate analyses (age, sex, iris colour, Ki-67 expression, emissary canal invasion, AJCC 8th edition stage, scleral extension, location of anterior margin, vortex vein invasion, cell type, necrosis, and nuclear BAP1 expression). Results were reported as hazard ratios (HR) with 95% confidence intervals (CI). Statistical significance was defined as p < 0.05.

All analyses were performed using IBM SPSS Statistics version 29.0 (IBM Corp., Armonk, NY, USA).

### Ethics statement

Ethical approval was granted by the Research Ethics Committee of the University Clinical Hospital of Valladolid. The study was conducted by the principles of the 2013 Declaration of Helsinki.

## Results

### Population characteristics

A total of 150 patients were included in the study, all of whom were White Caucasian. There were 89 men and 61 women, and the median age was 65 years. The epidemiological and clinical characteristics of the study population are reported in **Table 1**. The median age at diagnosis of uveal melanoma was 65 years; given this, 65 years was chosen as a cut-off for splitting the sample by age, yielding a similar number of patients in each group. Notably, nearly half (46%) of the patients had brown eyes, while 32% had an intermediate phenotype and 22% had blue/grey eyes. Regarding tumor size, the largest tumor diameter was 10–16 mm in 44% and >16 mm in 49% of cases. Most tumors were classified as IIIA (39%) and stage IIB (31%). Nearly half (47%) of the tumors were mushroom-shaped. The scleral extension was observed on imaging in 7% of patients. Only two patients had metastases at the time of diagnosis.

**Table 1:** Epidemiological, clinical and histopathological uveal melanoma characteristics of the study population.

n (%)			
(n=150)			
Sex		Location of anterior margin	
Male	89 (60)	Choroid	129 (86)
Age		Ciliary body	21 (14)
At enucleation (y), median	65	CELL TYPE	
Age ≤ 65 years	75 (50)	Spindle A, Spindle B	46 (31)
Age > 65 years	75 (50)	Mixed	64 (43)
Eye		Epithelioid	39 (26)
Right	80 (53)	Mitotic count	
Left	70 (47)	≤ 5	123 (82)
Iris colour		> 5	27 (18)
Brown	67 (46)	Ki-67 expression	
Green-hazel/intermediate	44 (32)	< 15%	96 (64)
Blue/grey	32 (22)	≥ 15%	54 (36)
Largest diameter (mm)		Optic nerve invasion	17 (11)
< 10	10 (7)	Vortex vein invasion	4 (3)
10 -16	66 (44)	Emissary canal invasion	33 (24)
>16	74 (49)	Scleral extension	
AJCC stage 8 th edition		None or innermost layers of sclera	134 (90)
IIA	22 (15)	Within sclera or to scleral surface	4 (3)
IIB	46 (31)	Extrascleral extension without presumed residual tumour in the orbit	6 (4)
IIIA	59 (39)	Extrascleral extension with presumed residual tumour in the orbit	4 (3)
IIIB	19 (13)	Inflammation	
IIIC	4 (3)	None to minimal	138 (93)
Tumour shape		Marked	34 (23)
Dome	60 (42)	Necrosis	
Mushroom	71 (47)	No	100 (68)
Diffuse	16 (11)	<10%	31 (21)
Scleral extension		≥ 10%	16 (11)
Yes	11 (7)	Nuclear BAP1 staining	
Metastases		Low grade	75 (50)
Yes	2 (1)	High grade	75 (50)

The histopathological characteristics are summarized in **Table 1**. The most common anatomical location of the anterior margin was the choroid (86%). The most common cell type was mixed (43%). Overall, 82% of patients showed low mitotic activity (≤5 mitotic figures per-40 high-power fields) and 64% low Ki-67 expression (<15%). Emissary canal, optic nerve, and vortex vein invasion were seen in 33 (24%), 17 (11%), and 4 (3%) patients, respectively. The majority of patients (n = 134, 90%) showed no scleral involvement or only involvement of the innermost layers of the sclera. Moderate or marked inflammation was observed in 10 patients (7%), moderate or marked pigmentation in 111 patients (75%) and necrosis affecting ≥10% of the tumor tissue in 16 patients (11%). The nuclear expression of BAP1 was observed to be of low grade in approximately 50% of the patients and of high grade in the remaining 50%, demonstrating a balanced distribution.

### Clinicopathological analyses

**Table 2** summarizes the clinicopathological characteristics of enucleated eyes with uveal melanoma, stratified by high or low grade nuclear BAP1 immunostaining. Statistically significant associations were observed between nuclear BAP1 grade and patient age (p=0.005), scleral extension (p=0.028), anterior tumour margin location (p=0.036), and tumour necrosis (p=0.006). Specifically, low grade BAP1 expression was more common in patients older than 65 years, and in those with scleral extension. Tumours with high grade BAP1 staining also more frequently displayed a choroidal anterior margin and less necrosis. No significant associations were found with sex, eye laterality, iris colour, tumour diameter, cell type, mitotic count, Ki-67 index, optic nerve or emissary canal invasion, tumour shape, metastasis, inflammation, or pigmentation.

**Table 2:** The clinicopathological features of enucleated eyes with low or high grade nuclear bap1 staining.

	Nuclear BAP1 staining		*p* Value
	High grade	Low grade	
Sex			0.618
Male	46 (61 %)	43 (57 %)	
Female	29 (39 %)	32 (43 %)	
Age			0.002
Age ≤ 65 years	47 (63 %)	28 (37 %)	
Age > 65 years	28 (37 %)	47 (63 %)	
Eye			0.326
Right	37 (49 %)	43 (57 %)	
Left	38 (51 %)	32 (43 %)	
Iris colour			0.245
Brown	37 (51 %)	30 (42 %)	
Green-hazel/intermediate	23 (32 %)	21 (30 %)	
Blue/grey	12 (17 %)	20 (28 %)	
Largest diameter (mm)			0.140
< 10	4 (5 %)	6 (8 %)	
oct-16	39 (52 %)	27 (36 %)	
>16	32 (43 %)	42 (56 %)	
AJCC stage 8 th edition			0.011
IIA	15 (20 %)	7 ( 9%)	
IIB	29 (39 %)	17 (23 %)	
IIIA	25 (33 %)	34 (45 %)	
IIIB	5 (7 %)	14 (19 %)	
IIIC	1 (1 %)	3 (4 %)	
Tumour shape			0.264
Dome	29 (39 %)	34 (45 %)	
Mushroom	35 (47 %)	36 (48 %)	
Diffuse	11 (15 %)	5 (7 %)	
Scleral extension			0.028
No	73 (97 %)	66 (88 %)	
Yes	2 (3 %)	9 (12 %)	
Metastases			0.245
No	72 (97 %)	75 (100 %)	
Yes	2 (3 %)	0 (0 %)	
Location of anterior margin			0.036
Choroid	69 (92 %)	60 (80 %)	
Ciliary body	6 (8 %)	15 (20 %)	
Cell type			0.118
Spindle A, Spindle B	32 (43 %)	20 (27 %)	
Mixed	29 (39 %)	38 (50 %)	
Epithelioid	14 (19 %)	17 (23 %)
Mitotic count			0.228
≤ 5	59 (79 %)	64 (85 %)	
> 5	16 (21 %)	11 (15 %)	
Ki-67 expression			0.307
< 15%	45 (60 %)	51 (68 %)	
≥ 15%	30 (40 %)	24 (32 %)	
Optic nerve invasion			0.071
No	63 (84 %)	70 (93 %)	
Yes	12 (16 %)	5 (7 %)	
Emissary canal invasion			0.808
No	55 (76 %)	57 (78 %)	
Yes	17 (24 %)	16 (22 %)	
Scleral extension			1
None or innermost layers of sclera Within sclera or to scleral surface	69 (93 %)	69 (93 %)	
Extrascleral extension without presumed residual tumour in the orbit. Extrascleral extension with presumed residual tumour in the orbit	5 (7 %)	5 (7 %)	
Inflammation			0.242
None to minimal	67 (91 %)	72 (96%)	
Moderate	5 (7 %)	1 (1 %)	
Marked	2 (3 %)	2 (3 %)	
Pigmentation			0.07
None to minimal	25 (33 %)	13 (17 %)	
Moderate	36 (48 %)	42 (56 %)	
Marked	14 (19 %)	20 (27 %)	
Necrosis			0.006
No	60 (80 %)	40 (56 %)	
<10%	9 (12 %)	22 (30 %)	
≥ 10%	6 (8 %)	10 (14 %)	

### Overall survival

**Figure 3:**
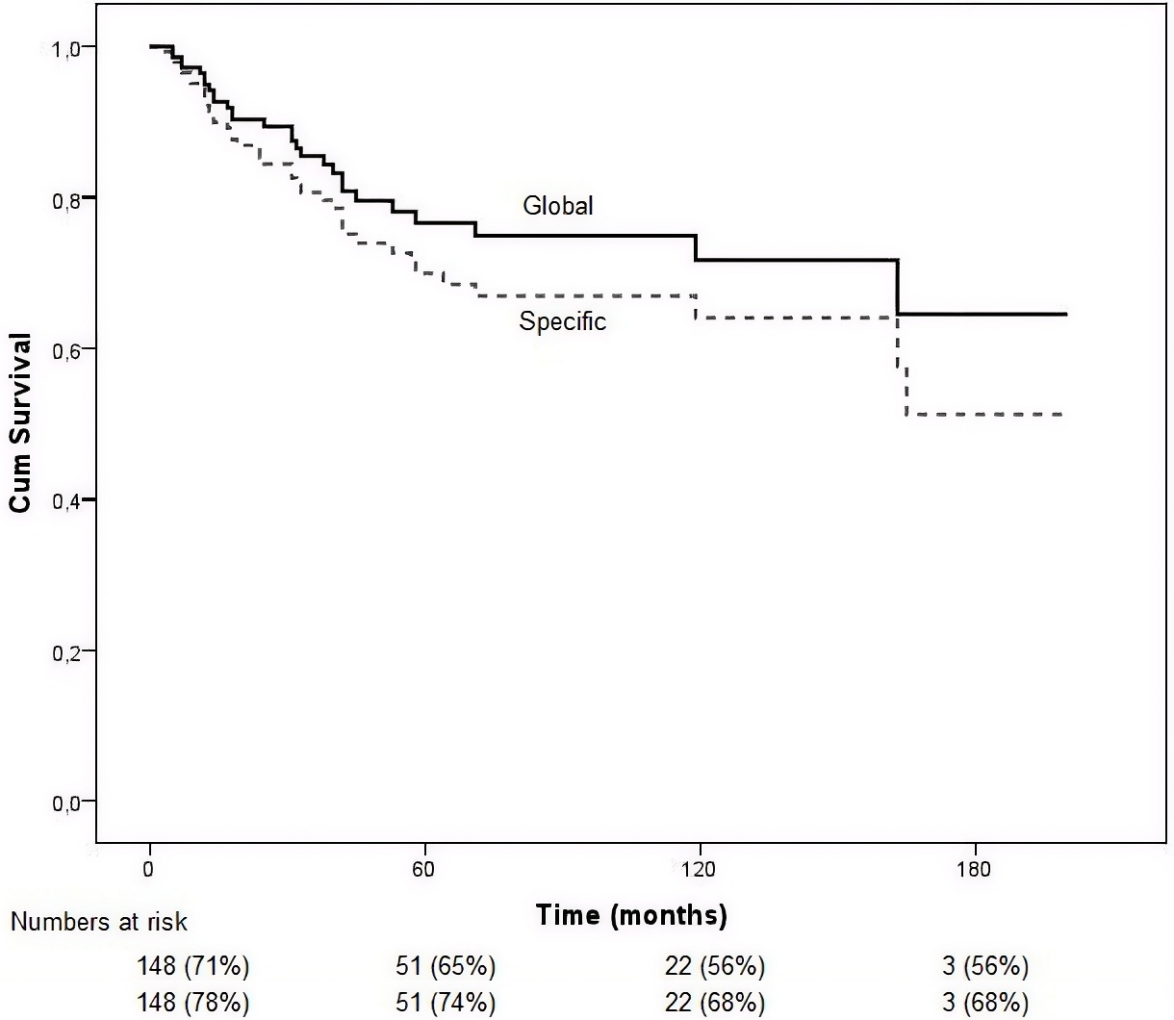
Kaplan-Meier estimates of overall and specific survival in the sample of 148 enucleated uveal melanoma patients.

During a mean follow-up of 54 months, 39 deaths from melanoma and other causes were recorded. The mean overall survival was 137.3 months (95% CI: 121.2–153.4). **Figure 3** shows the follow-up of the cohort: of the 148 patients at baseline, 93 survived to 30 months, 51 to 60 months, 22 to 120 months, and 3 patients remained alive at 180 months (15 years).

### Specific survival

28 patients died due to metastatic uveal melanoma. The mean disease-specific survival was 152.5 months (95% CI: 137.0–168.0). The estimated probability of disease-specific survival was 76% at 30 months, 75% at 60 months, and 72% at 90 months, with a progressive decline thereafter (**Figure 3**).

### Prognostic factors and survival

Kaplan–Meier survival analysis was performed to assess the prognostic impact of various histopathological features in patients with uveal melanoma.

**Figure 4:**
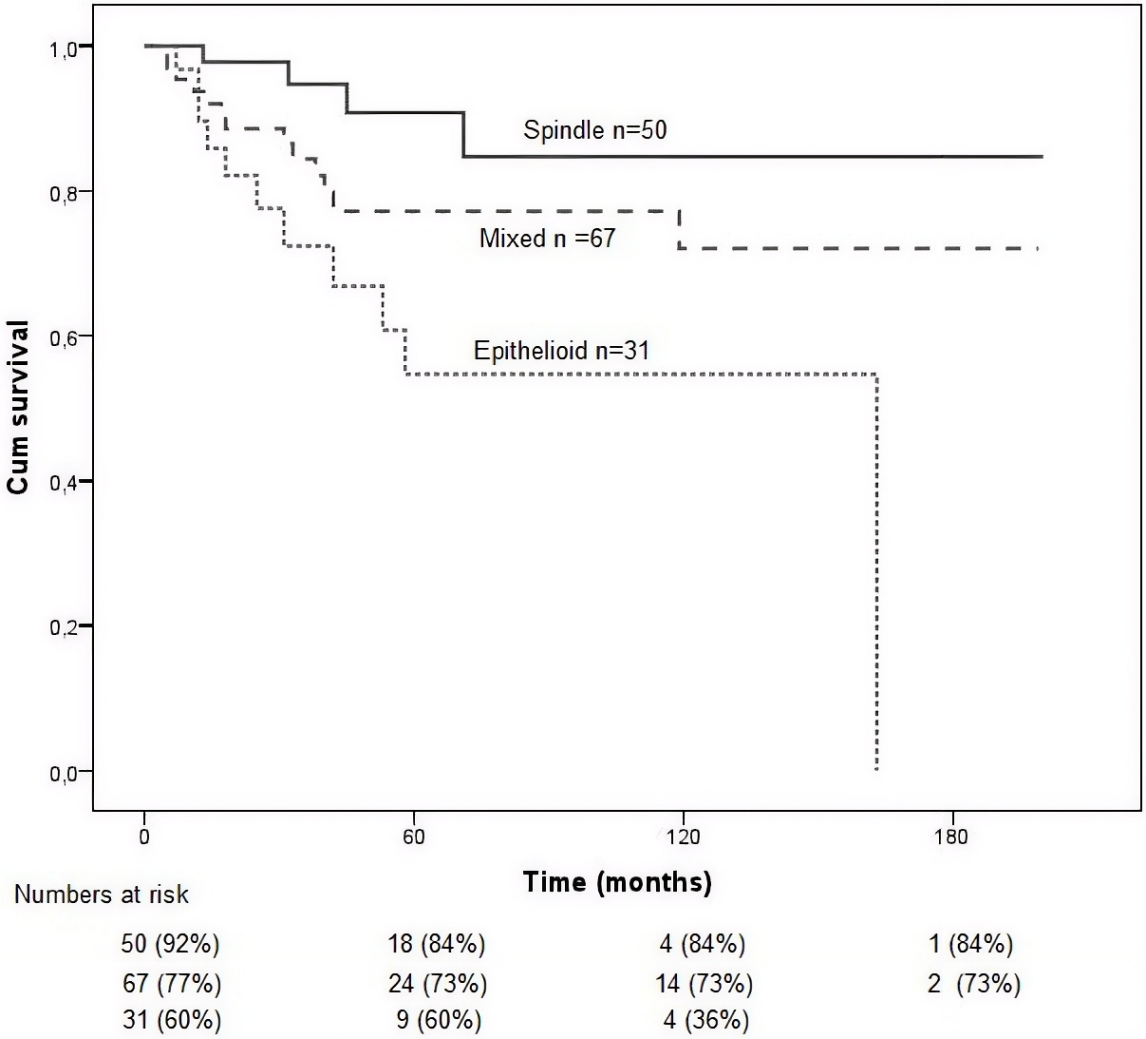
Kaplan–Meier survival curves for the study population stratified by cell type (epithelioid, spindle, and mixed). Overall survival was calculated from the time of diagnosis to the last follow-up or death. Differences between groups were assessed using the log-rank test. Patients with epithelioid cell type showed the poorest prognosis (*p* = 0.008).

Cell type (**Figure 4**) was a significant predictor of survival. Patients with spindle cell melanomas (types A and B) had the longest survival times, followed by those with mixed cell types, while epithelioid tumors were associated with the poorest prognosis (*p* = 0.008).

**Figure 5:**
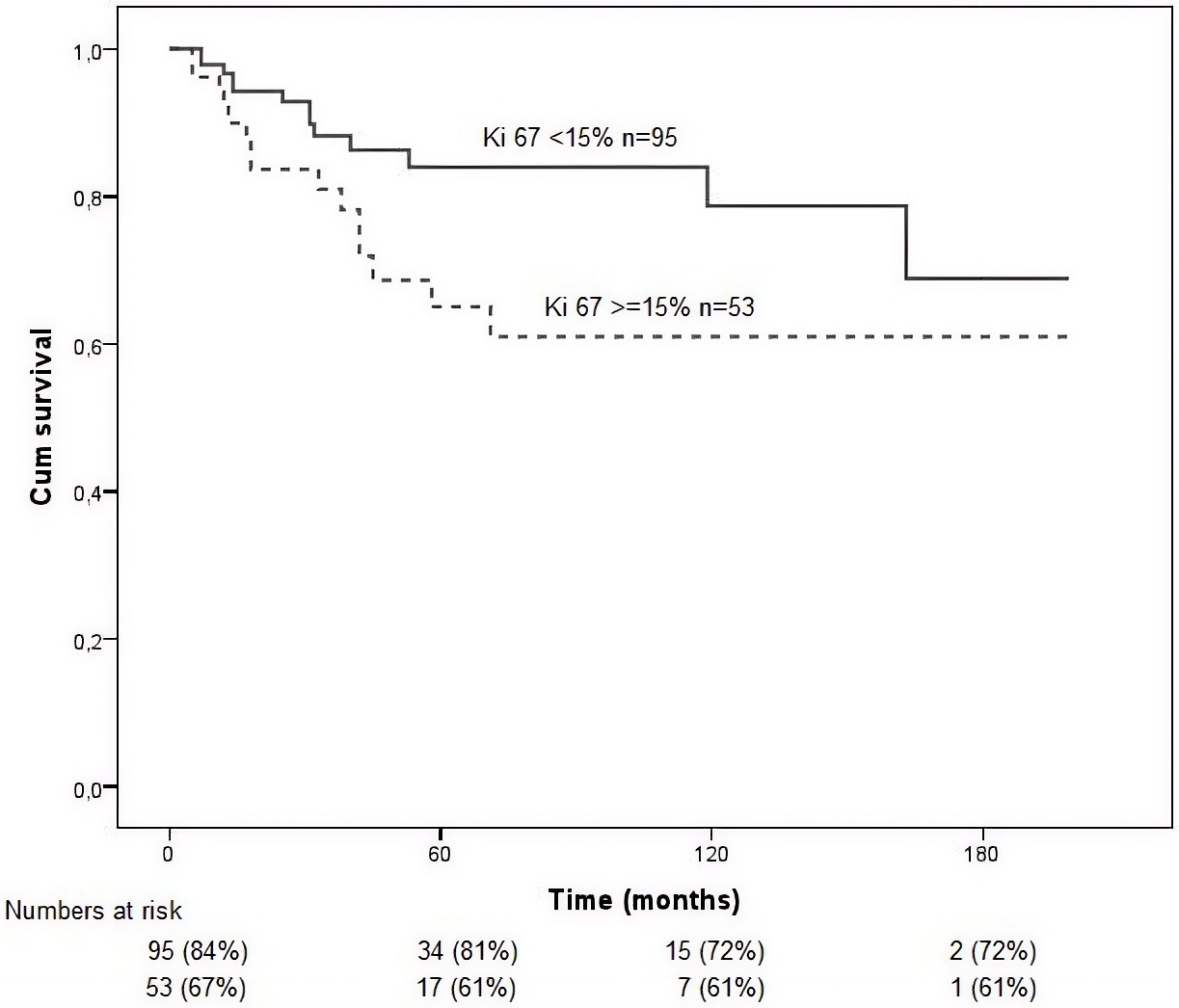
Kaplan–Meier survival curves for the study population stratified by Ki-67 expression (<15% vs ≥15%). Overall survival was calculated from the time of diagnosis to death or last follow-up. Differences between groups were assessed using the log-rank test. Elevated Ki-67 expression (≥15%) was associated with reduced overall survival (*p* = 0.035).

Elevated Ki-67 expression (≥15%; **Figure 5**) correlated with reduced overall survival, confirming its role as a marker of proliferative activity and aggressiveness (*p* = 0.035).

Vortex vein involvement was associated with poorer survival (*p* = 0.001).

**Figure 6:**
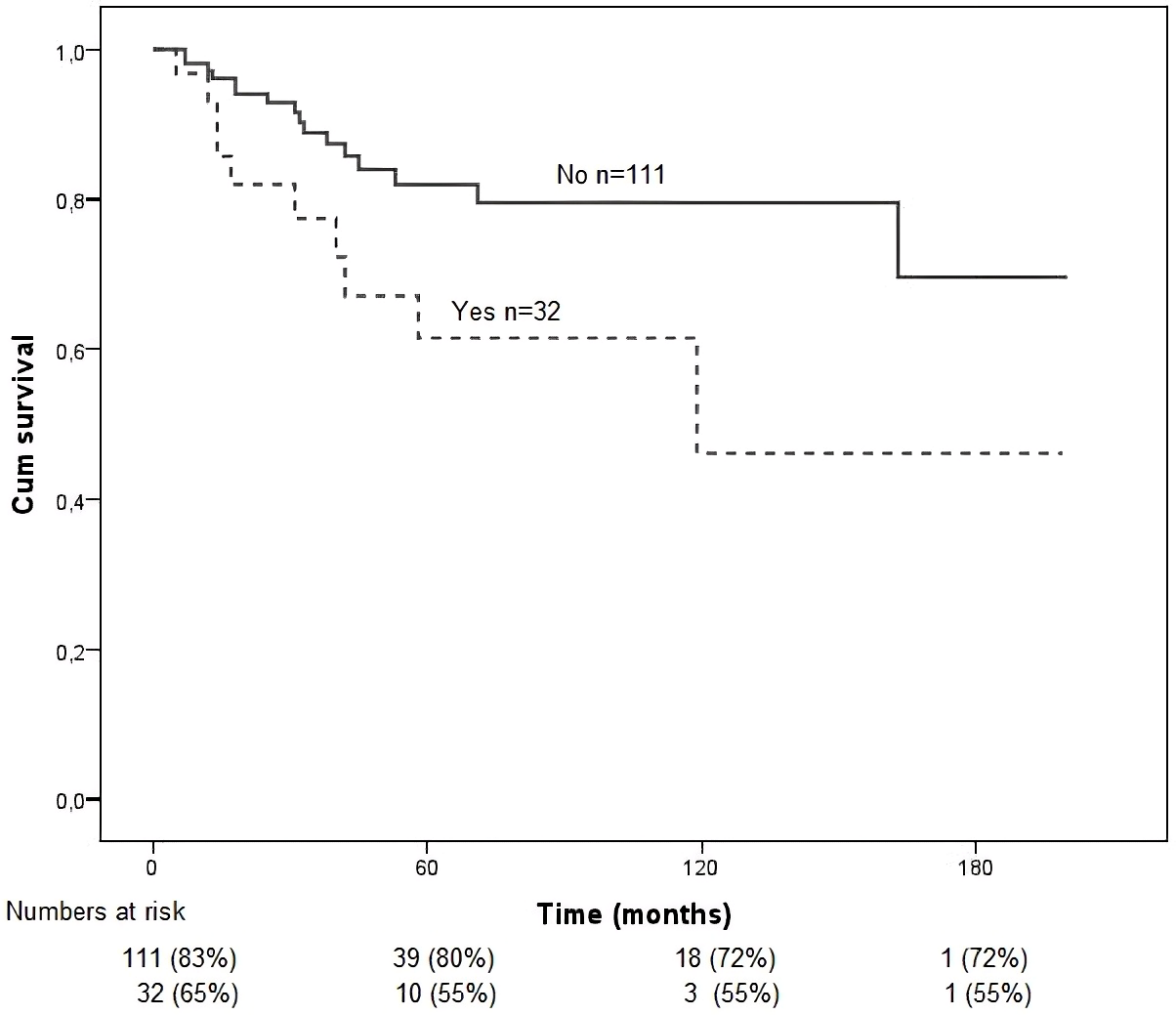
Kaplan–Meier survival curves for the study population stratified by the presence or absence of emissary canal invasion. Overall survival was calculated from the time of diagnosis to death or last follow-up. Differences between groups were assessed using the log-rank test. Emissary canal invasion was associated with worse overall survival (*p* = 0.021).

Emissary canal invasion (**Figure 6**) showed significantly worse overall survival compared to those without this feature (*p* = 0.021). This finding underscores the adverse prognostic impact of emissary canal involvement, likely reflecting more aggressive or advanced local tumor behavior.

**Figure 7:**
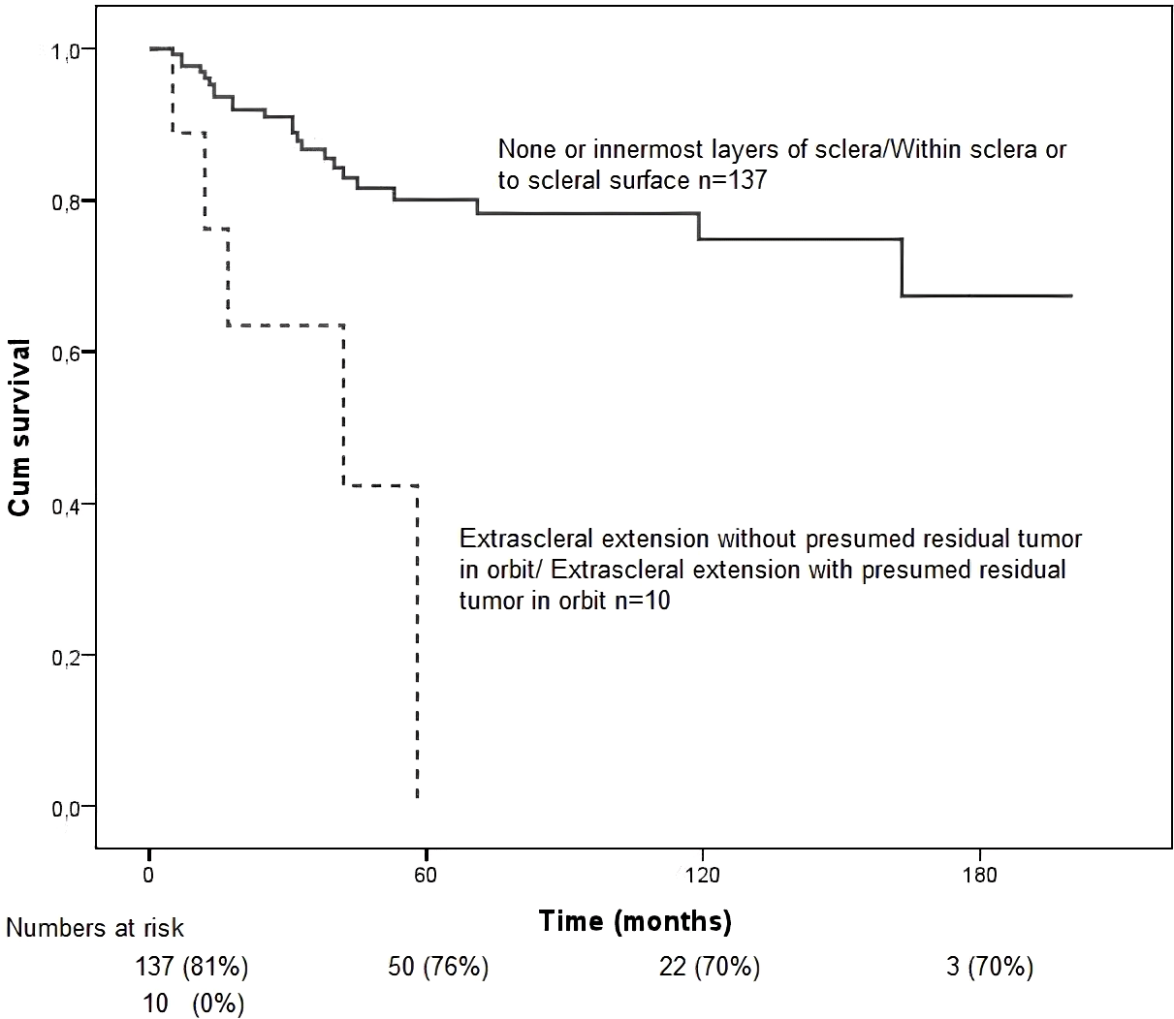
Kaplan–Meier survival curves for the study population stratified by scleral extension (presence vs absence). Overall survival was calculated from the time of diagnosis to death or last follow-up. Differences between groups were assessed using the log-rank test. Scleral extension was associated with poorer survival outcomes (*p* = 0.000).

Patients with tumours with extrascleral extension involving presumed residual tumor in the orbit had the poorest survival outcomes (*p* = 0.000, **Figure 7**). Survival improved progressively in cases with less extensive involvement, with significantly better outcomes observed in tumors confined to the innermost layers of the sclera.

**Figure 8:**
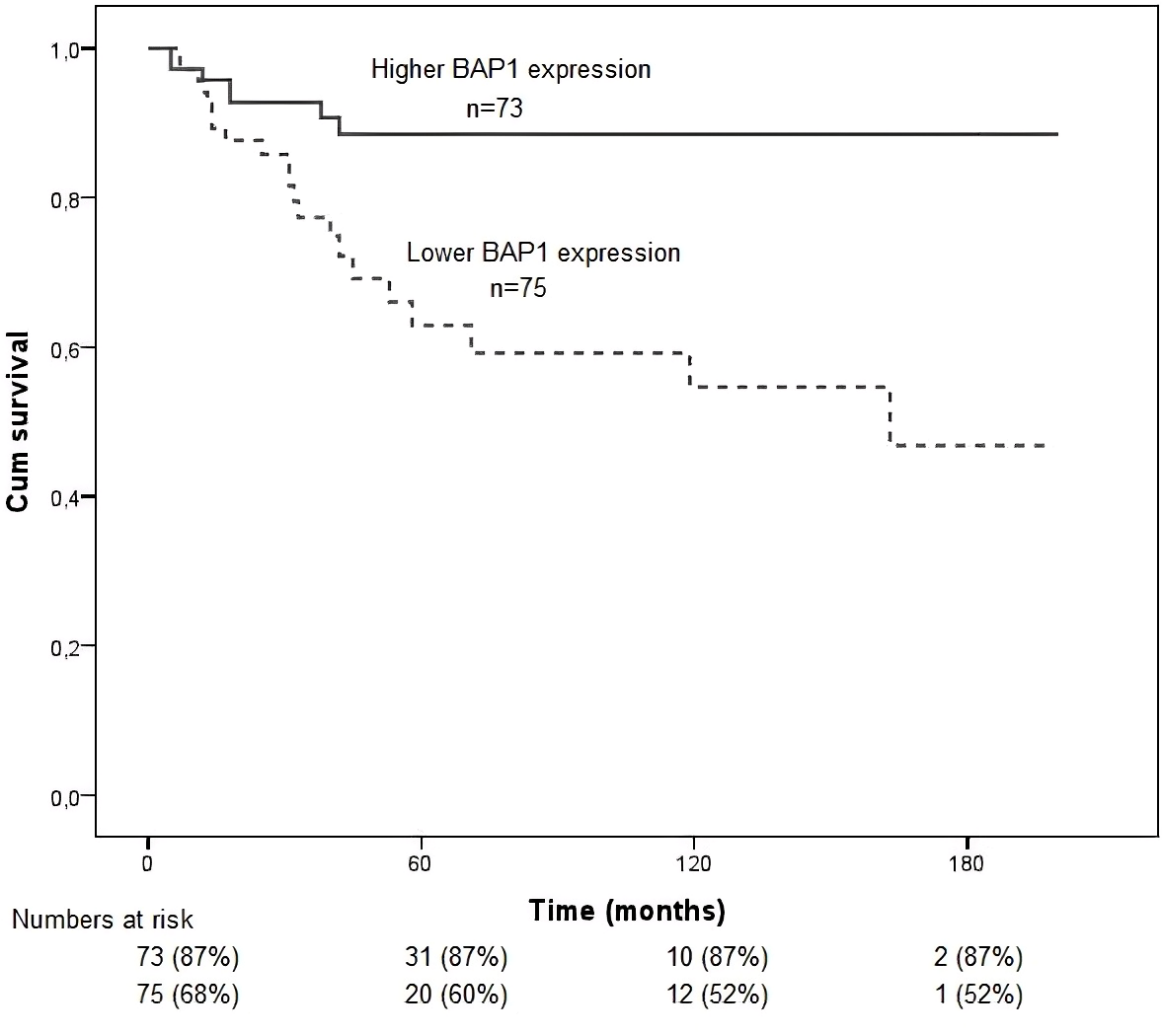
Kaplan–Meier survival curves for the study population stratified by BAP1 expression (high vs low nuclear staining). Overall survival was calculated from the time of diagnosis to death or last follow-up. Differences between groups were assessed using the log-rank test. Low BAP1 expression was associated with worse prognosis (*p* = 0.002).

Low nuclear BAP1 staining had a worse prognosis (*p* = 0.002; **Figure 8**).

### Multivariate analyses

In the multivariate Cox regression analysis, low grade staining of nuclear BAP1 was significantly associated with a poorer prognosis in patients with uveal melanoma (HR = 2.72; 95% CI: 1.04–7.11; *p* = 0.041), indicating that tumours exhibiting low BAP1 expression conferred approximately a 2.7-fold increased hazard compared to those with high grade staining. Additionally, invasion of vortex veins was an independent adverse prognostic factor, with a hazard ratio of 4.55 (95% CI: 1.18–17.53; *p* = 0.028). Extrascleral extension further increased the risk of unfavorable outcome nearly fivefold (HR = 4.73; 95% CI: 1.64–13.62; *p* = 0.004). Regarding cellular morphology, the epithelioid subtype was significantly associated with a worse prognosis compared to the fusiform subtype (HR = 4.12; 95% CI: 1.26–13.47; *p* = 0.019), whereas the mixed subtype did not reach statistical significance (HR = 1.69; 95% CI: 0.50–5.69; *p* = 0.398). These results highlight that low nuclear BAP1 expression, vortex vein invasion, extrascleral extension, and epithelioid cell type are independent predictors of poor clinical outcome in uveal melanoma (**Table 3**).

**Table 3:** Multivariate survival analysis.

		*p* Value	Hazard ratio	95% confidence interval for hazard ratio	
				Lower	Upper
Nuclear BAP1 staining		0, 041	2, 723	1, 042	7, 113
Vortex vein invasion		0, 028	4, 546	1, 179	17, 526
Scleral extension		0, 004	4, 729	1, 642	13, 617
Cell type	Spindle A, Spindle B (reference)	0, 031			
	Mixed	0, 398	1, 689	0, 501	5, 691
	Epithelioid	0, 019	4, 124	1, 262	13, 474
AJCC stage 8th edition	IIA	0.011			
	IIB	0.442	1.882	0.375	9.435
	IIIA	0.232	2.511	0.554	11.373
	IIIB	0.007	8.730	1.804	42.247
	IIIC	0.108	7.310	0.645	82.805

## Discussion

This study represents the largest and most mature prospective clinical case series of patients enucleated for posterior uveal melanoma from Spain, reporting the longest follow-up, with all cases managed within the same Adult Intraocular Tumours Unit under a standardized clinical and histopathological protocol. Our study adds robust evidence to the current literature on BAP1 immunohistochemistry in uveal melanoma, particularly due to its large sample size, long-term follow-up, and the use of a standardized, prospectively designed database. With 150 cases included and a median follow-up exceeding four years, this is, to our knowledge, the largest prospective cohort assessing the prognostic implications of BAP1 protein expression in a real-world population treated at a national referral center.

An additional strength of this cohort is its demographic homogeneity, as all included patients were White Caucasian. The prognostic role of BAP1 has not previously been evaluated in such a large, homogeneous population with extended follow-up. This is of particular interest given that survival outcomes in this subgroup appear slightly more favorable compared with some previously published international series (9), raising the possibility that the prognostic impact of BAP1 expression may differ across populations.

The main limitations of the study include the fact that all tumors were treated by enucleation and that most were large, which may limit generalizability to smaller tumors or eye-preserving strategies. This introduces a potential selection bias, as the cohort is enriched for more advanced disease requiring enucleation, and may not fully represent the entire spectrum of uveal melanoma patients. Only two patients presented with metastasis at the time of diagnosis, which precludes robust statistical analysis of associations involving metastatic disease and limits the statistical power for this variable. Another limitation relates to the BAP1 immunohistochemical cutoff used. We applied a 33% threshold for nuclear expression, consistent with the methodology reported by Herrspiegel et al. (30) where this cutoff was found to correlate with metastatic risk. Although the choice of threshold may influence result interpretation and comparability across studies, its use allows alignment with previously validated prognostic frameworks. Previous studies have consistently demonstrated the association between loss of nuclear BAP1 expression and metastatic risk in uveal melanoma. However, most published series have been limited by relatively small sample sizes, short follow-up, or highly selected patient populations. Szalai et al. (31) examined 40 enucleated UM specimens and found a significant association between low nuclear BAP1 expression and metastatic progression. However, the relatively small cohort and lack of long-term outcomes limited the generalizability of their findings. Similarly, See et al. (32) analyzed 30 patients over a 10-year period and demonstrated a significant correlation between low nuclear BAP1 expression and GEP class 2 tumors, with a positive predictive value for metastasis of 69%. While these findings are aligned with ours, their limited sample size and the use of gene expression profiling—which is not universally available—highlight the importance of developing accessible, cost-effective prognostic tools such as BAP1 immunohistochemistry.

Our results are consistent with these observations while substantially extending them through the inclusion of a larger cohort and longer follow-up, allowing for a more reliable assessment of metastasis-free survival. Importantly, our study reflects routine clinical practice over an extended time period, encompassing patients diagnosed and treated before the widespread implementation of molecular prognostic assays. This enhances the external validity and generalizability of our findings, particularly in healthcare settings where advanced molecular testing is not universally available.

The findings of the present study also corroborate and expand upon prior Spanish data reported by Tabuenca del Barrio et al. (33), who conducted the first Spanish study on this topic. They evaluated 40 UM cases and reported a significant association between low nuclear BAP1 staining and worse metastasis-free survival. However, our study extends previous work by including a substantially larger and more diverse cohort, allowing for more robust statistical power and greater external validity.

Recent methodological approaches, such as digital image analysis, have explored intratumoral heterogeneity in BAP1 expression. Stålhammar & Grossniklaus (34), have provided additional insights into intratumoral heterogeneity in BAP1 expression. Their study found that such heterogeneity had no impact on prognosis, a finding that supports the reproducibility and reliability of BAP1 nuclear staining as a biomarker, even in the presence of variable expression patterns. However, their methodology focused on image-based metrics, whereas the present study results emphasize clinical outcomes, reinforcing the practical utility of conventional immunohistochemical evaluation in routine diagnostic settings and metastasis-free survival.

In a broader molecular context, Silva-Rodríguez et al. (35) reinforced the association between chromosome 3 monosomy, BAP1 mutations, and metastatic risk in UM. Their cohort included 46 patients and highlighted that 75% of those who developed metastases carried BAP1 mutations. While their study used molecular sequencing techniques, our findings demonstrate that immunohistochemical loss of nuclear BAP1 expression can serve as a practical surrogate, especially in centers lacking access to molecular diagnostics.

Furthermore, recent meta-analyses have emphasized the prognostic relevance of BAP1. For instance, a meta-analysis by Lamas et al. (36) included more than 500 patients from various studies and concluded that loss of nuclear BAP1 staining was significantly associated with metastasis and poor survival outcomes. Nonetheless, the heterogeneity of methods and cut-offs used across studies complicates direct comparisons. Our standardized protocol for IHC evaluation—including centralized pathology review and pre-defined scoring criteria—represents a methodological strength that helps address this limitation.

Another critical contribution of the present study is the demonstration that cytoplasmic BAP1 staining lacks prognostic value. This aligns with the observations from Szalai et al. (31), See et al. (32), and Tabuenca del Barrio et al. (33), who also reported no statistically significant associations between cytoplasmic BAP1 expression and metastatic risk. Our larger cohort further validates these findings and supports the notion that only nuclear localization of BAP1 is relevant to tumor suppression and metastatic behavior in UM.

In summary, our findings support the growing body of evidence that BAP1 nuclear immunohistochemistry is a strong prognostic biomarker in uveal melanoma. The large size of our cohort, the long and standardized follow-up, and the rigorous protocol used in database construction provide a high level of evidence. The present study results reinforce the clinical utility of BAP1 IHC as a surrogate for genetic profiling, offering an accessible, reproducible, and affordable alternative in routine diagnostic practice, particularly in resource-limited settings. Given the well-established role of chromosome 3 monosomy in the pathogenesis and prognosis of uveal melanoma, future studies should consider incorporating genetic analysis of chromosome 3 status alongside BAP1 immunohistochemistry. Combining these approaches could further refine prognostic accuracy and enhance risk stratification, potentially improving personalized patient management.

## Data Availability

The raw data supporting the conclusions of this article will be made available by the authors, without undue reservation.

## References

[1] Bishop KD Olszewski AJ . Epidemiology and survival outcomes of ocular melanoma: a population-based analysis. Int J Cancer. (2014) 134:2961–71. doi: 10.1002/ijc.28625. PMID: 24272143

[2] Chattopadhyay C Kim DW Gombos DS Oba J Qin Y Williams MD . Uveal melanoma: from diagnosis to treatment and the science in between. Cancer. (2016) 122:2299–312. doi: 10.1002/cncr.29727. PMID: 26991400 PMC5567680

[3] Virgili G Gatta G Ciccolallo L Capocaccia R Biggeri A Crocetti E . EUROCARE-5 Working Group: incidence of uveal melanoma in Europe. Ophthalmology. (2007) 114:2309–15. doi: 10.1016/j.ophtha.2007.01.032. PMID: 17498805

[4] Agarwala SS Eggermont AM O'Day S Zager JS . Metastatic melanoma to the liver: a contemporary and comprehensive review of surgical, systemic, and regional therapeutic options. Cancer. (2014) 120:781–9. doi: 10.1002/cncr.28480. PMID: 24301420

[5] Eskelin S Pyrhönen S Summanen P Hahka-Kemppinen M Kivelä T . Tumor doubling times in metastatic Malignant melanoma of the uvea: tumor progression before and after treatment. Ophthalmology. (2000) 107:1443–9. doi: 10.1016/s0161-6420(00)00182-2. PMID: 10919885

[6] Damato B Eleuteri A Taktak AF Coupland SE . Estimating prognosis for survival after treatment of choroidal melanoma. Prog Retin Eye Res. (2011) 30:285–95. doi: 10.1016/j.preteyeres.2011.05.003. PMID: 21658465

[7] Gelmi MC Houtzagers LE Wierenga APA Versluis M Heijmans BT Luyten GPM . Survival in patients with uveal melanoma is linked to genetic variation at HERC2 single nucleotide polymorphism rs12913832. Ophthalmology. (2025) 132:299–308. doi: 10.1016/j.ophtha.2024.09.001. PMID: 39245076

[8] Jager MJ Shields CL Cebulla CM Abdel-Rahman MH Grossniklaus HE Stern MH . Uveal melanoma. Nat Rev Dis Primers. (2020) 6:24. doi: 10.1159/000095118. PMID: 32273508

[9] Valencia Nieto P García Álvarez C García Lagarto ME Muñoz Moreno MF Diezhandino García P Saornil Álvarez MA . Clinical and histopathological prognostic factors in uveal melanoma in a large Spanish series of enucleated eyes. Acta Ophthalmol. (2025) 103:e332–40. doi: 10.1111/aos.17493. PMID: 40176715

[10] Nayman T Bostan C Logan P Burnier MN . Uveal melanoma risk factors: a systematic review of meta-analyses. Curr Eye Res. (2017) 42:1085–93. doi: 10.1080/02713683.2017.1297997. PMID: 28494168

[11] Singh AD Topham A . Incidence of uveal melanoma in the United States: 1973-1997. Ophthalmology. (2003) 110:956–61. doi: 10.1053/j.semdp.2015.10.005. PMID: 12750097

[12] Wu SN Qin DY Zhu L Guo SJ Li X Huang CH . Uveal melanoma distant metastasis prediction system: a retrospective observational study based on machine learning. Cancer Sci. (2024) 115:3107–26. doi: 10.1111/cas.16276. PMID: 38992984 PMC11462970

[13] Berus T Halon A Markiewicz A Orlowska-Heitzman J Romanowska-Dixon B Donizy P . Clinical, histopathological and cytogenetic prognosticators in uveal melanoma - a comprehensive review. Anticancer Res. (2017) 37:6541–9. doi: 10.21873/anticanres.12110 29187428

[14] Kaliki S Shields CL Shields JA . Uveal melanoma: estimating prognosis. Indian J Ophthalmol. (2015) 63:93–102. doi: 10.4103/0301-4738.154367. PMID: 25827538 PMC4399142

[15] Harbour JW Onken MD Roberson ED Duan S Cao L Worley LA . Frequent mutation of BAP1 in metastasizing uveal melanomas. Science. (2010) 330:1410–3. doi: 10.1126/science.1194472. PMID: 21051595 PMC3087380

[16] Jensen DE Proctor M Marquis ST Gardner HP Ha SI Chodosh LA . BAP1: a novel ubiquitin hydrolase which binds to the BRCA1 RING finger and enhances BRCA1-mediated cell growth suppression. Oncogene. (1998) 16:1097–112. doi: 10.1038/sj.onc.1201861. PMID: 9528852

[17] Nishikawa H Wu W Koike A Kojima R Gomi H Fukuda M . BRCA1-associated protein 1 interferes with BRCA1/BARD1 RING heterodimer activity. Cancer Res. (2009) 69:111–9. doi: 10.1158/0008-5472.can-08-3355. PMID: 19117993

[18] Masclef L Ahmed O Estavoyer B Larrivée B Labrecque N Nijnik A . Roles and mechanisms of BAP1 deubiquitinase in tumor suppression. Cell Death Differ. (2021) 28:606–25. doi: 10.1038/s41418-020-00709-4. PMID: 33462414 PMC7862696

[19] Bononi A Giorgi C Patergnani S Larson D Verbruggen K Tanji M . BAP1 regulates IP3R3-mediated Ca2+ flux to mitochondria suppressing cell transformation. Nature. (2017) 546:549–53. doi: 10.1038/nature22798. PMID: 28614305 PMC5581194

[20] Abdel-Rahman MH Pilarski R Cebulla CM Massengill JB Christopher BN Boru G . Germline BAP1 mutation predisposes to uveal melanoma, lung adenocarcinoma, meningioma, and other cancers. J Med Genet. (2011) 48:856–9. doi: 10.1136/jmedgenet-2011-100156. PMID: 21941004 PMC3825099

[21] Bott M Brevet M Taylor BS Shimizu S Ito T Wang L . The nuclear deubiquitinase BAP1 is commonly inactivated by somatic mutations and 3p21.1 losses in Malignant pleural mesothelioma. Nat Genet. (2011) 43:668–72. doi: 10.1038/ng.855. PMID: 21642991 PMC4643098

[22] Pilarski R Byrne L Carlo MI Hanson H Cebulla C Abdel-Rahman M . BAP1 tumor predisposition syndrome. In: Adam MP Feldman J Mirzaa GM Pagon RA Wallace SE Amemiya A , editors.GeneReviews®. University of Washington, Seattle, Seattle (WA (2016). p. 1993–2025. 27748099

[23] Walpole S Pritchard AL Cebulla CM Pilarski R Stautberg M Davidorf FH . Comprehensive study of the clinical phenotype of germline BAP1 variant-carrying families worldwide. J Natl Cancer Inst. (2018) 110:1328–41. doi: 10.1093/jnci/djy171. PMID: 30517737 PMC6292796

[24] Haugh AM Njauw CN Bubley JA Verzì AE Zhang B Kudalkar E . Genotypic and phenotypic features of BAP1 cancer syndrome: a report of 8 new families and review of cases in the literature. JAMA Dermatol. (2017) 153:999–1006. doi: 10.1001/jamadermatol.2017.2330. PMID: 28793149 PMC5710339

[25] Koopmans AE Verdijk RM Brouwer RW van den Bosch TP van den Berg MM Vaarwater J . Clinical significance of immunohistochemistry for detection of BAP1 mutations in uveal melanoma. Mod Pathol. (2014) 27:1321–30. doi: 10.1038/modpathol.2014.43. PMID: 24633195

[26] Scott IU Murray TG Hughes JR . Evaluation of imaging techniques for detection of extraocular extension of choroidal melanoma. Arch Ophthalmol. (1998) 116:897–9. doi: 10.1001/archopht.116.7.897. PMID: 9682702

[27] Rao PK Barker C Coit DG Joseph RW Materin M Rengan R . NCCN guidelines insights: uveal melanoma, version 1.2019. J Natl Compr Canc Netw. (2020) 18:120–31. doi: 10.6004/jnccn.2020.0007 32023525

[28] McLean IW Foster WD Zimmerman LE Gamel JW . Modifications of Callender's classification of uveal melanoma at the Armed Forces Institute of Pathology. Am J Ophthalmol. (1983) 96:502–9. doi: 10.1016/j.ajo.2018.08.025. PMID: 6624832

[29] Amin MB Greene FL Edge SB Compton CC Gershenwald JE Brookland RK . The Eighth Edition AJCC Cancer Staging Manual: continuing to build a bridge from a population-based to a more "personalized" approach to cancer staging. CA Cancer J Clin. (2017) 67:93–9. doi: 10.3322/caac.21388 28094848

[30] Herrspiegel C Kvanta A Lardner E Ramsköld Cabaca L Wells J Bartuma K . Nuclear expression of BAP-1 in transvitreal incisional biopsies and subsequent enucleation of eyes with posterior choroidal melanoma. Br J Ophthalmol. (2021) 105:582–6. doi: 10.1136/bjophthalmol-2020-316498. PMID: 32522791 PMC8005798

[31] Szalai E Wells JR Ward L Grossniklaus HE . Uveal melanoma nuclear BRCA1-associated protein-1 immunoreactivity is an indicator of metastasis. Ophthalmology. (2018) 125:203–9. doi: 10.1016/j.ophtha.2017.07.018. PMID: 28823399 PMC6173805

[32] See TR Stålhammar G Phillips S Grossniklaus HE . BAP1 immunoreactivity correlates with gene expression class in uveal melanoma. Ocul Oncol Pathol. (2020) 6:129–37. doi: 10.1159/000502550. PMID: 32258021 PMC7109388

[33] Tabuenca Del Barrio L Nova-Camacho LM Zubicoa Enériz A Martínez de Espronceda Ezquerro I Córdoba Iturriagagoitia A Borque Rodríguez-Maimón E . Prognostic factor utility of BAP1 immunohistochemistry in uveal melanoma: a single center study in Spain. Cancers (Basel). (2021) 13:5347. doi: 10.3390/cancers13215347. PMID: 34771510 PMC8582434

[34] Stålhammar G Grossniklau HE . Intratumor heterogeneity in uveal melanoma BAP-1 expression. Cancers (Basel). (2021) 13:1143. doi: 10.3390/cancers13051143 33800007 PMC7962103

[35] Silva-Rodríguez P Bande M Fernández-Díaz D Lago-Baameiro N Pardo M José Blanco-Teijeiro M . Role of somatic mutations and chromosomal aberrations in the prognosis of uveal melanoma in a Spanish patient cohort. Acta Ophthalmol. (2021) 99:e1077–89. doi: 10.1111/aos.14760. PMID: 33421325

[36] Lamas NJ Martel A Nahon-Estève S Goffinet S Macocco A Bertolotto C . Prognostic biomarkers in uveal melanoma: the status quo, recent advances and future directions. Cancers (Basel). (2021) 14:96. doi: 10.3390/cancers14010096. PMID: 35008260 PMC8749988

